# Opportunities Offered by Chiral η^6^-Arene/*N*-Arylsulfonyl-diamine-Ru^II^ Catalysts in the Asymmetric Transfer Hydrogenation of Ketones and Imines

**DOI:** 10.3390/molecules16075460

**Published:** 2011-06-28

**Authors:** Jiří Václavík, Petr Kačer, Marek Kuzma, Libor Červený

**Affiliations:** 1 Department of Organic Technology, Institute of Chemical Technology, Technická 5, CZ-166 28 Prague, Czech Republic; 2 Laboratory of Molecular Structure Characterization, Institute of Microbiology, *v.v.i.*, Academy of Sciences of the Czech Republic, Vídeňská 1083, CZ-142 20, Prague, Czech Republic

**Keywords:** asymmetric transfer hydrogenation, imines, ketones, chiral, molecular modelling

## Abstract

Methods for the asymmetric transfer hydrogenation (ATH) of ketones and imines are still being intensively studied and developed. Of foremost interest is the use of Noyori’s [RuCl(η^6^-arene)(*N*-TsDPEN)] complexes in the presence of a hydrogen donor (*i*-PrOH, formic acid). These complexes have found numerous practical applications and have been extensively modified. The resulting derivatives have been heterogenized, used in ATH in water or ionic liquids and even some attempts have been made to approach the properties of biocatalysts. Therefore, an appropriate modification of the catalyst that suits the specific requirements for the reaction conditions is very often readily available. The mechanism of the reaction has also been explored to a great extent. Model substrates, acetophenone (a ketone) and 6,7-dimethoxy-1-methyl-3,4-dihydroisoquinoline (an imine), are both reduced by this Ru catalytic system with almost perfect selectivity. However, in each case the major product is a different enantiomer (*S*- for an alcohol, *R*- for an amine when the *S*,*S*-catalyst is used), which demanded an in-depth mechanistic investigation. Full-scale molecular modelling of this system enabled us to visualize the plausible 3D structures of the transition states, allowing the proposition of a viable explanation of previous experimental findings.

## 1. Introduction

It is a well-known fact that optically pure compounds play an inimitable role in various branches of the chemical industry, with pharmaceutical and fine chemical production processes being the most prominent ones [1,2]. Nowadays, enantioenriched chiral substances can be obtained in various ways, e.g., *via* chiral resolution of a racemic mixture, enzymatic catalysis, isolation of chiral compounds from natural resources, or catalytic asymmetric synthesis [3].

Chiral separation of enantiomers from a racemate prepared by standard organic techniques is still a widely-used method in asymmetric synthesis [4,5,6,7], mainly for economic reasons. However, chiral resolution brings unfavourable additional steps into the chain of synthetic reactions. Moreover, the other enantiomer is often of no value after the resolution.

Enzymatic catalysis stands in contrast to this classical method [8], exploiting the chirality imprinted in species of natural origin. Notwithstanding the superb selectivity achievable by means of enzymatic catalysis, the use of enzymes is often disadvantageous due to the strict conditions required to maintain their integrity and efficacy. 

Catalytic asymmetric synthesis stands somewhere “in between” when compared to chiral resolution techniques and enzymatic reactions, particularly in terms of cost and efficacy. Without a doubt, it embodies one of the pillars of modern chemistry. Although it is endowed with an exquisite toolbox for the synthesis of optically pure substances [9,10,11], much still remains to be explored.

Among the most frequently used techniques for enantioselective synthesis is asymmetric hydrogenation, already a 40 year old method that exploits an astonishing variety of organometallic complex catalysts [12,13,14]. The asymmetric bias of the reaction is generally reached by employing chiral ligands, which provide the necessary chiral environment around the central metal atom. The first successful ligands capable of stereoselective reductions were DIOP [15,16] (a bisphosphine developed by Kagan and Dang in 1971) and CAMP [17] (a monophosphine introduced by Knowles and co-workers in 1972). Using rhodium-based precursors, these ligands formed *in situ* catalytic complexes able to hydrogenate unsaturated carboxylic acids, giving amino acids of good optical purity. It was indeed a huge breakthrough in chemistry, as Knowles justifiably pointed out [13]: “(...) this was the first time ever that anyone had obtained enzyme-like selectivity with a man-made catalyst! Never in our wildest imagination did we think a structure versus activity study would converge so quickly to a product with commercial potential. CAMP was our sixth candidate. As I look back from this perspective, I don’t think that even we were emotionally equipped to realize what we had done.”

During the 40 years of asymmetric hydrogenation development, a myriad of ligands and catalysts have emerged, giving >99% *ee*s in reductions of C=C, C=O and C=N double bonds present in a variety of substrates. The majority of the ligands developed for asymmetric synthesis are those based on the phosphorus atom, which was thoroughly covered in a recent monograph [18]. In addition to these, hybrid P,N ligands [19,20] have also been developed. This review, however, focuses on a defined group of ligands containing nitrogen donor atoms.

To date the original Noyori-type η^6^-arene/*N*-arylsulfonyl-1,2-diphenylethylenediamine-ruthenium(II) catalysts have performed well in asymmetric transfer hydrogenations (ATH) of various groups of substrates [21,22,23,24,25,26,27,28] and have found numerous practical applications [29,30,31,32,33,34,35,36,37]. Naturally, a number of reviews have been published concerning both these remarkable catalytic hydrogenation systems [7,38,39,40,41,42,43] and their reaction mechanisms [44,45,46,47]. It should be pointed out that these complexes have also been used for asymmetric hydrogenations (AH) [48,49], which represents an extraordinary feature since both mechanisms (ATH and AH) are usually not achievable using a common catalyst. This review examines the ATH of imines and ketones using Noyori’s extraordinary η^6^-arene/*N*-arylsulfonyl-1,2-diphenylethylenediamine-ruthenium(II) catalysts and discusses some mechanistic considerations based on the latest findings. Hence, [RuCl(η^6^-arene)(amino alcohol)] species, which were investigated at the same time as the [RuCl(η^6^-arene)TsDPEN] complexes, will not be covered, although they have also been tested by Noyori for the reduction of aromatic ketones [50]. Another analogous group of ligands comprises tosylated diaminocyclohexane (TsCYDN) [51] and its derivatives. Although these ligands are very similar to the DPEN-type auxiliaries, they are beyond the scope of this review.

In the first instance, these catalytic complexes were used in homogeneously catalyzed ATH of ketones and imines in organic media [21]. Although these initial experiments proved the indisputable significance of the newly-discovered catalysts (see Section 2.1.), they suffered from difficult catalyst separation, which is a common issue in homogeneous catalysis. Consequently the chiral product, which was often synthesised for biological uses, contained expensive and environmentally unfriendly ruthenium compounds. Therefore, successive developments in this area have focused on greener applications of the catalysts in hand, which in particular meant their immobilization or the replacement of the solvent with water or ionic liquids. In Section 2.2. to Section 2.4., some examples are given on how this can be accomplished when unmodified catalysts are used in practice. However, the majority of cases have dealt with changes to the catalyst structure so as to improve its properties for a desired use. This large group of derivatives is covered in subchapter 3 which is further structured thematically. Hand in hand with these overall improvements came the possibility of catalyst recycling, which is of foremost practical importance.

The last subchapter of this review outlines the knowledge of the reaction mechanisms through which the ATH of ketones and imines operate under different circumstances. While the reaction mechanism is very well characterised in the case of ketones, a different concept had to be devised for the ATH of imines. The mechanistic pathways further change upon using different hydrogen donor molecules, or conducting the reactions in aqueous solutions.

## 2. Original η^6^-Arene/*N*-sulfonyldiamine-Ru^II^ Catalysts

### 2.1. The Pioneering Works on η^6^-Arene/N-sulfonyldiamine-Ru^II^ Catalysts

**Figure 1:**
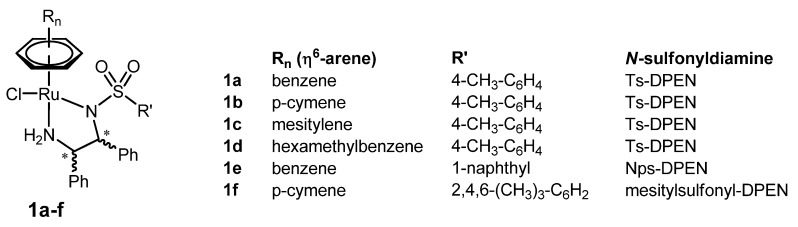
The basic set of η^6^-arene/*N*-sulfonyldiamine-Ru^II^ catalysts.

Beginning in 1995, Noyori’s η^6^-arene/*N*-arylsulfonyl-1,2-diphenylethylenediamine-ruthenium(II) catalysts **1(a-f)** [52,53,54,55] (Figure 1) emerged. These complexes were able to accelerate the hydrogenations of a wide range of aldehydes, ketones and imines with great selectivity [7,21,56]. This subsection briefly treats the original Ru^II^ complexes in selected examples of their original applications.

The Ru^II^ complexes are composed of two moieties: the η^6^-arene ligand and the chiral diamine auxiliary ligand. Benzene [53], *p*-cymene [53,55] and mesitylene [52,54,55] were first applied as the η^6^-arene ligands and shortly afterwards, hexamethylbenzene was tested as well [26]. The chiral diamine moiety was originally represented by *N*-tosyl-1,2-diphenylethylene-1,2-diamine (Ts-DPEN), *N*-(naphthalene-1-sulfonyl)-1,2-diphenylethylene-1,2-diamine (Nps-DPEN) and *N*-(2,4,6-trimethylphenylsulfonyl)-1,2-diphenylethylene-1,2-diamine (mesitylsulfonyl-DPEN). 

The original ligand combinations are outlined in Table 1 along with their performance data. Catalyst **1a** was found to be highly active in some cases, probably due to its η^6^-benzene ligand (entries 1 *vs*. 6). It was also described as efficacious in the ATH of 1-phenyl-3,4-dihydroisoquinoline derivatives [23], which proved unfeasible when using e.g., **1b**. Perhaps the most frequently used catalyst is **1b**, equipped with η^6^-*p*-cymene and TsDPEN, which has been shown to be able to efficiently hydrogenate imines and ketones with very high enantioselectivity (entries 2 and 3, refs. [53,57]). Catalyst **1c** was mainly used in the ATH of ketones, furnishing even higher *ee*s than **1b** (entries 4 and 5, refs. [52,54]). Usage of catalyst **1d** has been rather limited due to its lower activity. For instance, although it was demonstrated that **1d** accelerated the hydrogenation of benzaldehyde-*1*-*d* (entry 6), this reaction catalyzed by **1a** or **1b** proceeded up to 20 times faster with a very similar *ee* [26]. Catalyst **1e** has not been used very often in practice, owing to its very low solubility in most organic solvents [23]. However, similarly to **1a,** it was reported to be able to catalyze the ATH of 1-phenyl-3,4-dihydroisoquinoline derivatives (entry 7, ref. [53]). Above all, catalyst **1f** was tested in the ATH of 1-substituted 3,4-dihydroisoquinolines with very good enantioselectivity (entry 8, ref. [53]).

Basically, there are two ways to use such a complex as a catalyst: generation *in situ* or use of a preformed complex. Preparation of the catalyst *in situ* involves reacting the [RuCl_2_(η^6^-arene)]_2_ dimer with the corresponding *N*-arylsulfonyl-diphenylethylenediamine immediately before the hydrogenation, without further purification. This approach was actually applied in the first published work [52], where [RuCl_2_(η^6^-mesitylene)]_2_ and (*S*,*S*)-TsDPEN (Ru:TsDPEN = 1:2 molar) were heated at 80 °C for 20 min and then used in the reduction of aromatic ketones. For example, hydrogenation of acetophenone in such a manner gave (*S*)-1-phenylethanol in 97% *ee* and 95% yield (Entry 5 in Table 1). Preparing the catalyst this way may substantially help in overcoming certain difficulties, such as: (1) the true catalytic complex is not commercially available (whereas the precursors are); (2) the complex isolation is difficult or infeasible; or (3) the complex is poorly soluble. Preparation of the complex also involves a recrystallization step, which may thus be avoided if problematic.

On the other hand, usage of a preformed complex can sometimes be more convenient in practice, as only one compound is used in the catalyzed reaction. Additionally, successful preparation of a crystalline Ru catalytic complex can allow its characterisation by single-crystal X-ray diffraction analysis, providing the molecular geometry, which can be used in molecular modelling amongst other applications. In the remainder of this section various applications of the type **1** catalysts are discussed.

### 2.2. ATH Catalyzed by ***1*** in Water

Reactions carried out in water are very desirable as they are “green chemistry compatible” – in fact, enzyme-catalyzed reactions in nature proceed exclusively in aqueous media. Generally, such reactions may either form homogeneous (*i.e.*, both catalyst and substrate are soluble in water) or heterogeneous (one of the components being insoluble in water) systems [58]. The substrate and/or the catalyst often have low water solubility and, in contrast, the hydrogen donor can be hydrophilic. Numerous methods of overcoming these difficulties have been published.

In 2004, the ATH system under discussion was first reported to be practicable in water with surprisingly good results for the hydrogenation of aromatic ketones catalyzed by **1b**. In this work [59], Wu, Xiao *et al.* showed that the reaction proceeded much faster in HCOONa/H_2_O than in the HCOOH/triethylamine azeotrope. While in the HCOONa/H_2_O system acetophenone was completely reduced within 2 h (entry 1 in Table 2), in the azeotrope it took a reaction time of 12 h to reach almost full conversion (entry 2). Their explanation of this observation was based on the assumption that **1b** was more soluble in the substrate (organic phase) than in water. Following this, they revealed a strong pH dependence of the ATH of aromatic ketones in water using **1b** and HCOOH/triethylamine [60], proposing two different catalytic cycles for the reaction. While under basic conditions they expected the standard metal-ligand bifunctional mechanism to take place [61], low pH values were connected with protonation of the amido nitrogen of the TsDPEN ligand, which caused its partial decoordination. This phenomenon explained the loss of activity of **1b** under acidic conditions. Optimal pH values were described as between pH 5 and 8, which was effectively maintained by a suitable aqueous solution of HCOOH/triethylamine. Remarkably, this optimization of the reaction conditions allowed use of S/C ratios as high as 10,000.

Catalyst **1b** has also been integrated into micelle-like microreactors created from cetyltrimethylammonium bromide (CTAB) [62], which provided lipophilic reaction compartments where the ATH process could be accelerated. The model substrate, acetophenone, was reduced in up to 98% *ee* (Table 2, entry 3) by this catalytic system. Interestingly, the catalyst was reusable 6 times, where *ee* remained at ~95% and the catalyst activity decreased with each run. In addition to mere CTAB, the authors also tested combinations of surfactants, namely sodium dodecyl sulfate (SDS) with CTAB, where they found optimal reaction conditions with the ratio of SDS:CTAB being 2:1.

A remarkable work was published in 2007 [63], where a mixture of polyethylene glycol (PEG) and water was successfully applied in the ATH of aromatic ketones catalyzed by **1b**, using HCOONa as the source of hydrogen (Table 2, entry 4). A mixture of PEG:H_2_O (9:1, v/v) was found to be the optimal reaction solvent, which allowed 14 repetitions of use without deterioration in enantioselectivity and with only slightly decreased reactivity. Using this method chiral 1-phenylethanol was obtained in 94-96% *ee* (S/C = 100, 40 °C, 15-20 h for consecutive runs 1-9, and 20-40 h for runs 10-15). After each run, the product was easily extracted with hexane and the polymeric phase washed with HCOOH to regenerate the sodium formate it contained. ICP analysis showed that >99.9% of **1b** remained in the PEG phase.

Wang *et al*. [64] developed a reaction protocol for the ATH of ketones with **1b** and HCOONa in a mixture of water/dichloromethane, which was rather problematic as the substrate and catalyst were more soluble in the organic solvent, whereas the HCOO^–^ anion was present in the aqueous phase. The reaction was thus limited by the transport through the phase boundary. Their solution to this problem was to create an emulsion system by applying ultrasonic irradiation and a surfactant tetrabutylammonium iodide (TBAI), which caused a significant increase in the surface area between the organic and hydrophilic phases. Notably, this method facilitated the ATH of solid ketones, which is often slow or unfeasible when conducted in neat water due to their low water solubility. The high surface area created by emulsions provided proper contact of all the reaction components and the ATH was thus accelerated.

These examples demonstrate that the Ru^II^ catalysts **1** can also be effectively used in aqueous media. However, most ATHs in water using **1** have been described in conjunction with structural modifications of the catalyst, which is discussed further in section 3.1. and section 3.2.

### 2.3. Attempts to Immobilize Unmodified Complexes ***1***

Heterogeneous catalysts are heavily favoured in industry as they can be easily separated from the reaction mixture and the resulting product does not contain any residual catalyst, *i.e.*, unwanted compound(s) of heavy metals, which are expensive and may be harmful to the environment. Unfortunately, the structure of catalyst **1** is not particularly suitable for heterogenization without any further modification. The only reactive groups of the species are Ru-Cl and NH_2_ of TsDPEN, which, however, directly participate in the ATH mechanism [61,65] and thus should not be occupied by the solid support. Therefore, examples of immobilization of the unmodified complexes **1** are very scarce.

In 2003, de Smet, Vankelecom, *et al*. [66] used selective polydimethylsiloxane (PDMS) membranes to separate catalyst **1b**, aiming for a flow arrangement of ATH. After determination of the optimal conditions (mixture of methanol/isopropanol (30:70)), the membrane enabled the substrate and product to permeate and, simultaneously, was able to retain **1b** completely. This way, catalyst phase and bulk phase were separated by the membrane. In the ATH of acetophenone, 4 consecutive runs at a constant *ee* (95%) were performed, however 5% leaching of Ru was observed. To the best of our knowledge, this interesting idea has not been developed since.

Šiklová and co-workers [67] attempted to immobilize **1b** in the channels of MCM-41 molecular sieve functionalized by (3-aminopropyl)triethoxysilane (APTES). Their supported catalyst was used in the ATH of a cyclic imine (1-methyl-3,4-dihydroisoquinoline) to give 89% *ee* (Table 3, entry 1), which was the same value as they obtained for the homogeneous system. However, the reaction rate was lower in the case of the supported catalyst. This observation was ascribed to diffusion processes occurring within the channels of the solid material.

A very interesting concept was developed by Li, Yang and co-workers [68], who encapsulated **1b** in a mesoporous “cage”, *i.e.*, in the pores of the SBA-16 material. This was done by narrowing the pore size of SBA-16 by silylation with diphenyldichlorosilane. This complex was able to perform the ATH of acetophenone with 93% *ee* (Table 3, entry 2) and was reported to be reusable at least 6 times, although with a gradual decrease in catalytic activity. The concept of a mesoporous nanocage represented by SBA-16 was also applied in different catalytic systems [69,70,71].

### 2.4. ATH Catalyzed by ***1*** in Ionic Liquids

Ionic liquids are a desirable medium for conducting catalyzed reactions as they are ranked amongst the green solvents and in addition, they can facilitate catalyst recycling. A few examples of their use in ATH with **1** have been published.

In 2005 [72], the ATH of acetophenone was examined using **1a** in the ionic liquid 1-butyl-3-methylimidazolium hexafluorophosphate, also known as [bmim][PF_6_]. The reaction, employing HCOOH/triethylamine, gave 1-phenylethanol in 93% *ee* (96% conv., 24 h). The recycling is discussed in section 3.4.

The performance of **1b** in nine different ionic liquids was examined by Joerger *et al*. in 2006 [73]. Although the catalytic activity was found to be lower than under Noyori’s original conditions, high *ee*s (up to 97%) were obtained for the ATH of acetophenone. The catalyst could be recycled no more than 4 times, despite employing various methods (bulb-to-bulb distillation of the product, extraction).

Huťka and Toma [74] optimized the reaction conditions for the ATH of a variety of ketones in several ionic liquids (ethylmethylimidazolium ethylsulfate, ECOENG500, [bmim][BF_4_]), where aryl alkyl ketones were reduced with the highest enantioselectivity. Their results further showed a very small dependence of *ee* upon temperature.

## 3. Modifications of the η^6^-Arene/*N*-sulfonyldiamine-Ru^II^ Complexes

Perhaps the most interesting feature of asymmetric catalysis is the modularity of catalytic complexes. On the basis of the first successful catalytic systems, numerous alternative species are derived which mimic or exceed the properties of the original complex [75]. This basic idea has inspired many researchers to explore possible modifications of known catalysts, which would lead to improved catalytic behaviour of these species. Herein we present an overview of direct modifications to the structure of catalyst **1**.

The nature of the Noyori’s η^6^-arene/*N*-arylsulfonyl-1,2-diphenylethylenediamine-ruthenium(II) homogeneous catalyst **1** for the ATH of imines and ketones allows us to modify both moieties of the compound, *i.e.*, the η^6^-arene ligand and the chiral diamine. The structure of the chelate diamine ligand (1) is responsible for the configuration of the major product enantiomer [61,76], while changing the ligand configuration readily changes the product configuration and (2) contributes to the asymmetric bias in enantioselective reactions [61]. However, the latter is mainly governed by the selection of the η^6^-arene ligand substituents, which affect the structure of transition states of the hydrogenation process and thus alter the reaction enantioselectivity [61,77]. Moreover, both the η^6^-arene ring and the *N*-sulfonyl fragment influence the catalyst activity [52,54,61,77].

Thus, successful ligand modification is not a trivial process owing to the complicated factors governing the catalyst activity and capability of asymmetric induction in hydrogenation reactions. In this text, the variations of **1** discussed are divided into several sections, according to the purpose of the modifications. Therefore, structural alterations leading to improvements e.g., in catalyst immobilization or ATH in water and ionic liquids are discussed. However, due to the large number of derivatives of **1**, the scope of this review is limited and hence examples of straight-forward modifications of **1** (see e.g., [78,79,80,81,82,83]) will not be discussed in this work. Likewise, Wills’ well-known tethered complexes (see e.g., [84,85,86,87]) are not covered as they have been reviewed elsewhere [41,88].

### 3.1. Modifications of **1** Facilitating ATH in Aqueous Media

**Figure 2:**
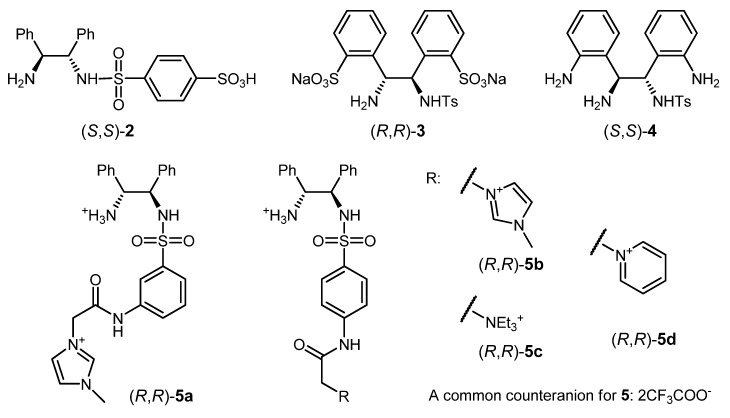
Modifications of TsDPEN used in ATH in water.

Small alterations of the diamine ligand in **1** led to the development of noteworthy catalytic systems enabling the implementation of ATH in water. This topic has also been reviewed recently [41,89,90]. In general, the derivatives generally bear a polar functional group which increases their water solubility. The structures are depicted in Figure 2.

The first hydrophilic modifications of catalytic complex **1b** appeared in 2001, when Bubert *et al*. [91] introduced a sulfonic acid group in the *para-* position of the *N*-arylsulfonyl fragment of TsDPEN. The novel ligand **2** furnished chiral 1-phenylethanol in 94% *ee* in the ATH of acetophenone using [RuCl_2_(η^6^-*p*-cymene)]_2_ (Table 4, entry 1). At the same time, the ligand was also used in rhodium- and iridium-based catalysts, showing both higher activity and selectivity in the ATH of acetophenone under identical conditions [92].

In 2003, Deng and co-workers disclosed a TsDPEN ligand modification bearing two *ortho*-sulfonate groups (**3**, Scheme 2) [93]. The ligand was easy to prepare and water-soluble. The first experiments on the ATH of ketones with **3** were carried out using the [RuCl_2_(η^6^-*p*-cymene)]_2_ ligand and sodium formate as a hydrogen source instead of HCOOH/triethylamine. In addition, the presence of sodium dodecyl sulfate (SDS) or 15-crown-5 was found to be necessary to increase the reaction rate. 

Under these conditions, a range of aromatic ketones were successfully reduced, including *α*-bromoacetophenone, which was complicated when the HCOOH/triethylamine azeotrope was used [97]. Chiral 1-phenylethanol was obtained in 95% *ee* (entry 2) and the ATH of *α*-bromoacetophenone in a H_2_O/CH_2_Cl_2_ mixture proceeded with 94% *ee* (entry 3).

The sulfonated ligand **3** was subsequently applied to the ATH of imines and iminiums under similar conditions [94]. However, when SDS was used as a surfactant both yield and enantioselectivity decreased. Therefore, other surfactants had to be tested and cetyltrimethylammonium bromide (CTAB) gave the best results (entry 4). A variety of cyclic imines (isoquinolines, *β*-carbolines, *N*-sulfonylimines) were successfully reduced this way, although in the case of some acyclic imines, this catalytic system has been found to be unsuitable. When the **3**-based catalyst was tested for recycling, the enantioselectivity remained constant and yield slightly decreased with repeated catalyst use. The catalytic system developed was also utilized for the hydrogenation of *tetra*-alkyl-substituted iminium substrates, which were found to be more reactive than their *tri*-alkylated imine analogues.

In 2007 Li, Deng *et al*. introduced an *o*,*o’*-aminated TsDPEN ligand **4** for ATH in water conducted without the presence of surfactants [95]. Although the catalyst, formed *in situ* with [Ru(η^6^-*p*-cymene)Cl_2_]_2_, was not overly active in the ATH of acetophenone using HCOONa (entry 5), its [Cp*RhCl_2_]_2_ analogue furnished 97% conversion under identical conditions. Many other aromatic ketones were also reduced using this complex, mostly with excellent enantioselectivity (>95%).

Zhou and co-workers [96,98] synthesized water-soluble ligands **5a**-**5d**, which, when combined with [Ru(η^6^-*p*-cymene)Cl_2_]_2_ and HCOONa, reduced aromatic ketones with great enantioselectivity (entries 6 and 7). Their water solubility was promoted by a quarternary ammonium group (imidazolium [98], or triethylammonium and pyridinium [96]) which was attached to the TsDPEN moiety. The ligands were tested for recycling (five or six consecutive runs) in the ATH of acetophenone, where their activity only slightly decreased and *ee* remained constant at 93% in the case of **5c** (100% conv., S/C = 100, 2-3 h, 40 °C) [96]. The **5a**/**5b** based catalysts showed great activity (quantitative conversions within 2 hours even after 6 runs), although erosion of *ee* was observed [98]. Ligands **5a** and **5b** were also used for ATH in ionic liquids [99] (see section 3.4.).

### 3.2. ATH Conducted on Immobilized Catalysts Derived from ***1***

A variety of approaches to the immobilization of **1** have emerged, offering solid catalysts applicable in heterogeneous ATHs of ketones and imines. This generally involved introduction of a functional group onto the aromatic rings of the diamine ligand, which provided the necessary linkage to the solid material.

Perhaps the first attempt at heterogenization of **1** was published in 1997 [100]. The TsDPEN ligand was replaced by *N*-[(4-vinylphenyl)sulfonyl]-1,2-diphenylethylene-1,2-diamine, which was subsequently copolymerized with styrene (1:10) into a linear polymer (**6a**), or with styrene and divinylbenzene (1:10:0.5) into a cross-linked polymer (**6b**). These two ligands were tested with [Ru(η^6^-benzene)Cl_2_]_2_, [Ru(η^6^-*p*-cymene)Cl_2_]_2_ and [Ir(COD)Cl_2_]_2_ precursors in the ATH of acetophenone using isopropanol as the hydrogen source. Although the iridium-based catalysts were both more active and selective (up to 94% *ee* at 96% yield after 48 h), their stability was disappointingly low. The ruthenium complexes were somewhat more stable (*i.e.*, reusable up to 4 times) but they gave significantly lower *ee*s and were less active (see Table 5, entry 1).

In 1998 [101], Bayston *et al*. immobilized **1** onto aminomethylated polystyrene (PS), with (**7b**) or without (**7a**) polyethylene glycol (PEG) spacer units, creating orange/red polymeric beads. The PEG-containing ligand **7b** was used with [Ru(η^6^-*p*-cymene)Cl_2_]_2_ in the ATH of acetophenone in a neat HCOOH/triethylamine mixture, and gave (*S*)-1-phenylethanol in 96.7% *ee* (Table 5, entry 3). Ligand **7a** was more active when cosolvents were used (DMF, CH_2_Cl_2_), giving the product in amazing > 99% *ee* (entry 2). However, both catalysts were only reusable twice.

In 2004, many contributions in the field of heterogeneous ATH with modified **1** appeared. Li, Xiao and co-workers attempted to attach the TsDPEN ligand *via* its phenyl rings to a polyethylene glycol support, creating an immobilized ligand **8** (PTsDPEN) [102]. When hydrogenating acetophenone in neat HCOOH/triethylamine mixture using **8** combined with [Ru(η^6^-*p*-cymene)Cl_2_]_2_, 94% *ee* was obtained (entry 4). Efforts were made to reuse the catalyst by precipitation with diethyl ether, however, only three runs were practicable due to a rapid loss of catalytic activity. The use of **8** was also investigated for the ATH of ketones in water using HCOONa as a hydrogen donor, which caused a huge increase in the reaction rate [103]. In the ATH of acetophenone using **8** with [Ru(η^6^-*p*-cymene)Cl_2_]_2_, 1-phenylethanol (*ee* 92%) was obtained in full conversion within 1 hour (entry 5). Furthermore, the PTsDPEN-based catalyst was found to be much more stable in the aqueous phase, which enabled more efficient catalyst recycling by precipitation with diethyl ether. The first 12 consecutive runs were shown to be feasible at a constant *ee* without a significant loss in activity. Ligand **8** therefore seemed to be highly attractive.

Liu, Tu *et al.* chose the *N*-arylsulfonyl group of TsDPEN as a linker to a solid support, which was represented by amorphous silica gel (in **9a**) and mesoporous silicas (MCM-41 in **9b** and SBA-15 in **9c**) [104,105]. These inorganic solids were selected for their high stability at high temperatures and in organic solvents. Surprisingly, cheap amorphous silica gel exceeded both MCM-41 and SBA-15 in performance. When conducting the ATH of acetophenone in neat HCOOH/triethylamine mixture with **9a** and [Ru(η^6^-*p*-cymene)Cl_2_]_2_ [104], the product was obtained in full conversion after 6 hours (Table 5, entry 6). When catalyst recycling was attempted, no more than five runs were feasible, which was caused by severe leaching of the ruthenium. The best results were obtained when *o*- and *m*-fluorinated acetophenones were tested for recycling where 10 consecutive runs were successfully accomplished (*ee* 93-94%, activity decreasing). However, as the activity of this catalyst in the HCOOH/triethylamine azeotrope was only moderate, HCOONa was also used for the ATH in water [105], with tetrabutylammonium bromide (TBAB) applied as an additive, which was chosen after screening a number of surfactants and phase transfer catalysts. The reaction rate was markedly accelerated; optically enriched 1-phenylethanol was obtained quantitatively within 2 hours and in 96% *ee* (entry 7). In terms of reusability the acetophenone was recycled 6 times, and 11 runs were achieved in the case of 4’-bromoacetophenone. Further experiments were carried out on these complexes [106], giving results consistent with the initial data.

**Figure 3:**
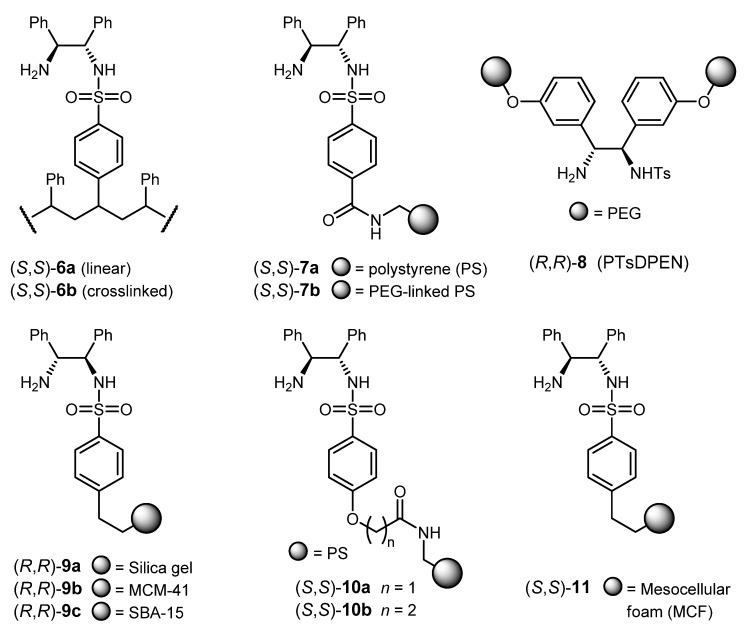
Immobilized TsDPEN-based ligands (I).

Two novel polystyrene-supported ligands **10a** and **10b** (Figure 3) were synthesized by attaching modified TsDPEN to aminomethylated polystyrene [107]. These were examined in the ATH step of the enantioselective synthesis of (*S*)-fluoxetine (entry 8). The final pharmaceutical product was obtained in 97% *ee* and contained less than 0.04 ppm of ruthenium.

**Figure 4:**
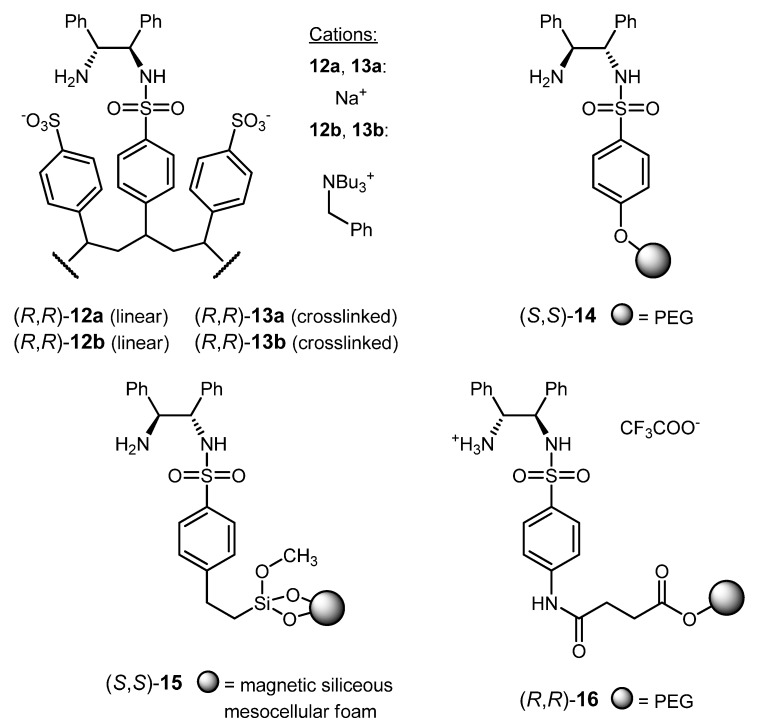
Immobilized TsDPEN-based ligands (II).

Another work dealing with the immobilization of **1b** onto an inorganic support was published in 2007 by Huang and Ying [108]. They compared silica gel and siliceous mesocellular foam (MCF), with the latter giving the best results when used for immobilization and subsequently used in ATH. Ligand **11** immobilized on MCF, used in combination with [Ru(η^6^-*p*-cymene)Cl_2_]_2_ and HCOOH/triethylamine, catalyzed the ATH of 6,7-dimethoxy-1-methyl-3,4-dihydroisoquinoline (entry 9) and several aromatic ketones with high yield and enantioselectivity. The ligand was reported to be reusable at least 6 times, although leaching of both Ru and ligand was observed. The group of Itsuno [109] modified the previously described polystyrene-supported ligand **6a** [100] by introducing *p*-styrenesulfonate instead of styrene in the copolymerization (ligands **12a** and **12b** in Figure 4).

Analogously, the cross-linked polymer (**13a**,**13b**) was obtained by copolymerization with *p*-styrenesulfonate and divinylbenzene. While the original ligands (**6a**,**6b**) were described as shrinking in water, these novel analogues were much more compatible with ATH in water thanks to the extra sulfonyl pendant groups. As cations, either Na^+^ (in **12a** and **13a**) or benzyltributylammonium (in **12b** and **13b**) were used, which allowed the modification of the lipophilic properties of the ligand. A very interesting observation was the enhanced enantioselectivity (98% *ee*) in the ATH of acetophenone using **12b** and **13b** with [Ru(η^6^-*p*-cymene)Cl_2_]_2_ and HCOONa (entries 10 and 11). The authors attributed this to the creation of microenvironments in the polymer structure causing stronger asymmetric bias. Reusability of the **13b**-containing catalyst was also addressed, where 5 repeated uses were reported. Two years later [114], this work was extended by two more pendant groups, carboxylate and alkanesulfonate, both again with Na^+^ or benzyltributylammonium cations. Nevertheless, neither of these surpassed their first ligands containing the arenesulfonate group. In 2009 [110], they further examined this concept in the ATH of imines. The sulfonylated polymers complemented with Na^+^ or benzyltributylammonium were found suitable for the ATH of cyclic imines in water utilizing HCOONa (entry 12).

Inspired by previous works on PTsDPEN (**8**) [102,103] (*vide supra*), Chan *et al*. introduced a polyethylene glycol chain in the *para*-position of the *N*-arylsulfonyl fragment of TsDPEN, creating PEG-BsDPEN (**14**) [111]. They found that the enantioselectivity of the acetophenone hydrogenation was slightly dependent on the amount of HCOONa. The catalyst was fairly active and recyclable, which could be seen from the 8th run where 96% conversion and 95% *ee* were reached within 4 hours (the first run is listed as entry 13). This ligand was then successfully applied in the syntheses of (*R*)-salmeterol [115] and (*R*,*R*)-formoterol [116].

Li *et al*. [112] extended the possibilities of immobilization in siliceous mesocellular foam (MCF) by grafting it with magnetic nanoparticles, aiming for an easily-separable heterogeneous catalyst (ligand **15** in Scheme 4). The area of applicability of nanoparticles in catalysis has been reviewed very recently [117,118,119,120]. The activity of the **15**-based catalyst was very good; for example, 6,7-dimethoxy-1-methyl-3,4-dihydroisoquinoline was reduced with 94% *ee* within 1.5 h, when [Ru(η^6^-*p*-cymene)Cl_2_]_2_ and HCOOH/triethylamine in dichloromethane were used (entry 14). It was successfully recycled nine times, with a slight erosion of *ee* (94-90%) and a decrease in activity caused by mechanical stirring and mild Ru leaching (11 mol% after nine runs). Several aromatic ketones were tested as well with good results achieved.

Another polyethylene glycol-supported ligand (**16**) was synthesized in 2010 [113]. In the ATH of acetophenone using [Ru(η^6^-*p*-cymene)Cl_2_]_2_ and HCOONa, 97% *ee* was observed (entry 15). However, the catalyst was not particularly suitable for recycling as only three consecutive runs could be performed due to a marked decrease in both activity and *ee*.

### 3.3. ATH Conducted on Dendrimeric Catalysts Derived from ***1***

Although the dendrimeric modifications of **1** partially overlap with the area of catalyst immobilization techniques treated in section 3.2., we set this group apart due to its exclusivity. The dendrimeric structures impart some unique properties to the catalysts, which are hardly achievable by other methods [121,122,123]. It is worth noting that the globular shape of the dendrimers allows for membrane filtration [124] or precipitation as a means of recycling the catalyst. Recent examples of dendrimeric catalysts derived from **1** are given above (Table 6).

**Figure 5:**
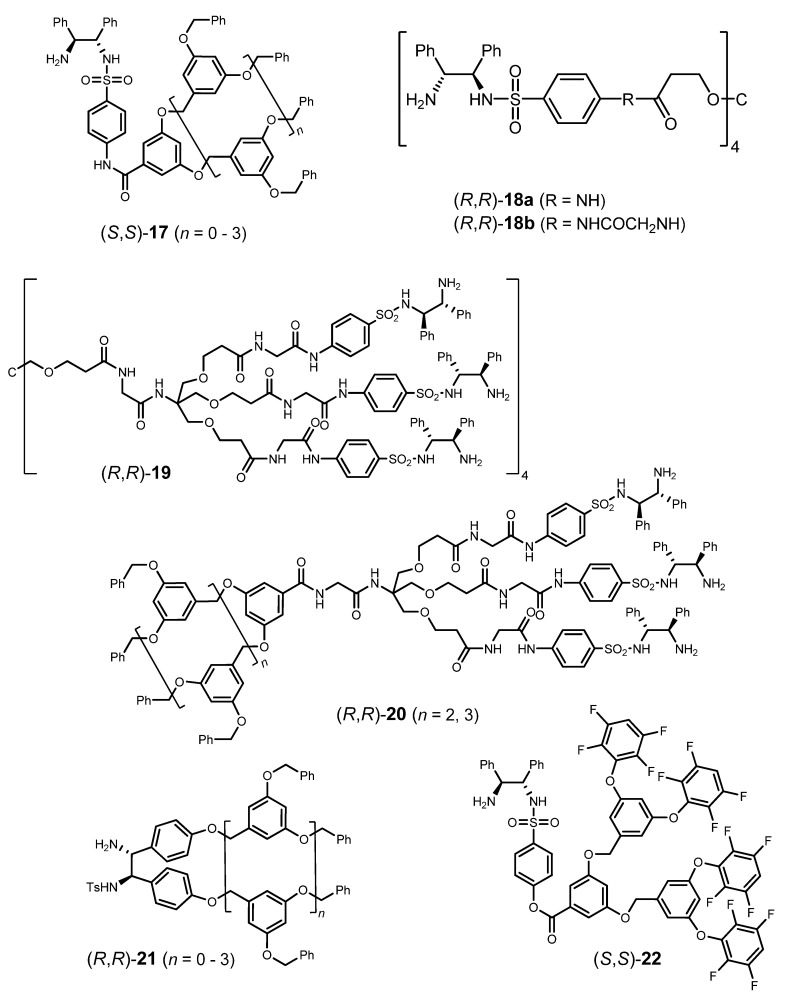
Dendritic TsDPEN-based ligands.

In 2001, Chen, Deng and co-workers [125] applied Fréchet’s polyether dendritic structures [126] to the TsDPEN ligand in the effort to find a recyclable catalyst. ATH of acetophenone using their dendritic ligands **17** (Figure 5) revealed they were recyclable up to five times, with constant enantioselectivity (*ee* > 96%) and decreasing catalyst activity.

The same group also developed a set of ligands where the TsDPEN units were located at the periphery of the dendrimer [57], believing that the diamines would be more accessible by the substrate molecules than in the former case [125], which would accelerate the reaction. For the first generation, tetrakis(2-carboxyethoxymethyl) methane was chosen as the core unit bearing 4 TsDPEN units connected *via* amide linkages, with (**18b**) or without (**18a**) glycine residues. As the second generation, a more complex ligand **19** bearing 12 diamine units was synthesized on the basis of Newkome’s cascade polymers [127]. In the ATH of acetophenone, the performance of **18a**, **18b** and **19** was compared with their monomeric analogues to find that both activity and enantioselectivity were almost identical for dendrimeric and monomeric ligands. These ligands proved applicable in the stereoselective hydrogenations of a number of aromatic ketones and imines.

Finally, Deng’s group synthesized unique hybrid dendritic catalysts **20** [128] by coupling the Fréchet- and Newkome-type ligands mentioned above. Catalyst recycling by precipitation was in the scope of this study, while it was shown that polyether dendrons of the Fréchet type [125] (**17**) were more suitable for recycling (up to six runs) due to their higher stability. In contrast, the diamine units in hybrid dendrimers **20** were located too far from the polyether moiety and the catalysts based on such dendrimers were less stable. The hybrid dendrimers therefore help to reveal that proximity of the diamines and the polyether dendrons was important for the catalyst stability.

Alternative dendrimeric ligands **21** were described in 2005, linking the diamine to the dendrons *via* the phenyl rings of TsDPEN [129]. The dendrimers were synthesized up to the third generation, and generations 1 and 2 were slightly more active than their amino-functionalized relatives [125]. However, the activity of the third generation dropped significantly, probably due to the steric inaccessibility of the active site by the substrate, caused by the bulky dendrons. The second generation was also tested for recyclability, although substantial leaching of the catalyst was observed (about 10 mol% in each run, determined by ICP) and therefore only four consecutive runs could be performed with conversion dropping by 5% each time. An interesting feature of these ligands was the possibility of changing the *N*-arylsulfonyl moiety, leading to higher enantioselectivities in some hydrogenations.

The group of Rothenberg developed a multilayered cylinder for membrane-like separation of the catalyst [130]. The cylinder was made of macroporous *α*-alumina covered by a thin layer of nanoporous *γ*-alumina. Because the steric bulk of **1b** had to be increased to prevent it penetrating the nanopores it was attached onto a third-generation dendrimer as reported by Deng (ligand **17**) [125]. Their membrane cup was capable of entrapping more than 99.7% of the Ru compounds after 3 days of stirring. Although this ceramic cylinder was tested with **17** (entry 6), it has the potential to be applied in a variety of catalyzed reactions where the catalytic species has sufficiently high molecular weight.

A fluorinated dendritic TsDPEN ligand **22** (FTsDPEN), which surpassed all previous dendritic catalysts based on **1** in terms of reusability, was introduced very recently [131]. **22** was tested with [Ru(η^6^-*p*-cymene)Cl_2_]_2_ in 30 consecutive runs of ATH of acetophenone using aqueous HCOONa and TBAI (S/C = 100, 40 °C). During the first 16 runs, *ee* was maintained between 93-95% and reaction time was 4-8 hours. From run 16 to 26, 12-24 hours were needed to achieve full conversion, while *ee* was within the range of 88-93%. The final runs showed a significant decrease in the catalyst activity. The recyclability of **22** was therefore very promising in comparison with the preceding studies [63,128], none of which achieved so many effective repeated uses.

### 3.4. ATH Conducted in Ionic Liquids Using Modified Catalysts ***1***

**Figure 6:**
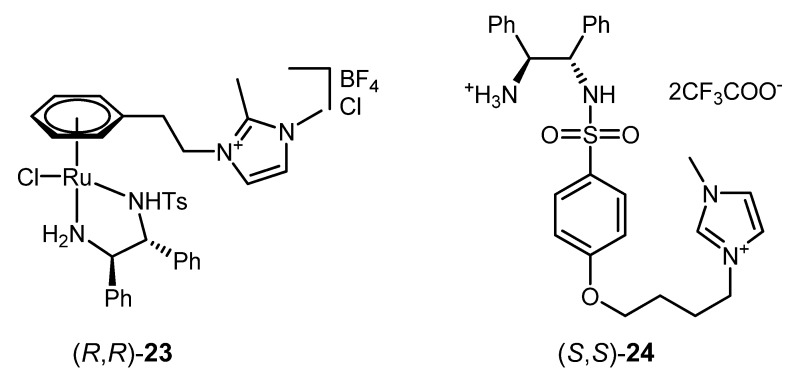
Ligands designed for ATH in ionic liquids.

This section covers the ligand modifications connected with usage in ionic liquids (Figure 6) and is closely linked with section 2.4. where unmodified complexes **1** are addressed.

Geldbach and Dyson [132] introduced an imidazolium group onto the η^6^-aromatic moiety of **1** creating its derivative **23**, which allowed immobilization in the ionic liquid 1-butyl-2,3-dimethylimidazolium hexafluorophosphate. Since KOH with isopropanol as a hydrogen source caused a sharp decrease in the catalytic activity, the HCOOH/triethylamine azeotrope was selected as an alternative. In this arrangement, the ATH of acetophenone was examined in comparison with original **1b**. The best recycling results were obtained with **1b** placed in ionic liquid, which afforded 5 consecutive runs with negligible loss in activity and >99% *ee* (Table 7, entry 1). After each run, the product was extracted with hexane and the ionic liquid containing the catalyst was dried under reduced pressure.

Ohta *et al*. [72] attached an imidazolium tag in the *para-* position of the TsDPEN ligand, creating **24**. They found that both **1a** (cf. section 2.4.) and the newly synthesized ligand, complexed to [Ru(η^6^-benzene)Cl_2_]_2_ *in situ*, were soluble in [bmim][PF_6_] under the reaction conditions and were suitable for recycling. The results obtained were very similar (5 consecutive runs, ~93% *ee*) but the imidazolium-containing ligand showed slightly better performance.

After successfully performing the ATH of aromatic ketones in water [98], Zhou *et al*. attempted to use their imidazolium-based ligands **5a** and **5b** (Scheme 2) also in ATH in the ionic liquid [bmim][PF_6_] [99]. They used the [Ru(η^6^-*p*-cymene)Cl_2_]_2_ precursor and HCOOH/triethylamine azeotrope for the ATH of acetophenone and obtained 1-phenylethanol in 97% *ee* (entry 3). Therefore, the reaction rate was about four times lower in comparison with the ATH in water [98]. Unfortunately, no more than 3 recycling runs were accomplishable due to a sharp decrease in catalytic activity.

### 3.5. Biomimetic Modifications of ***1***

**Figure 7:**
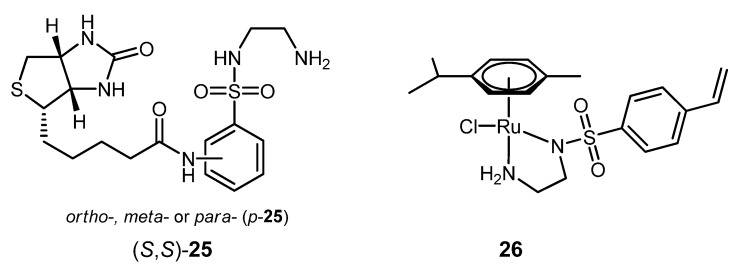
Ligands and complexes in biomimetic applications.

Some very interesing works have dealt with modifications of **1** aiming to approach enzyme-like performance. Although artificial metalloenzymes have been receiving increasing attention lately [133,134,135], not many works have focused upon the area of Noyori-type ATH of ketones and imines. The group of Ward *et al*. have done the most in this field [136,137,138,139,140]. In 2005 [136], they successfully anchored a Ru^II^ complex [(η^6^-arene)RuCl(**25**)], bearing a biotinylated achiral ligand (**25** in Figure 7), in a protein molecule of tetrameric streptavidin. The complex was tested in the ATH of acetophenone which revealed that the *p*-substituted ligand (*p*-**25**) was the best option. Interestingly, when η^6^-*p*-cymene was used in the complex, (*R*)-1-phenylethanol was produced, while with η^6^-benzene, the (*S*) enantiomer was observed. However, the *ee* values were modest and the catalytic activity was low. Therefore, they attempted to optimize the artificial enzyme by point mutations in streptavidin. It was shown that the mutations closest to the active site increased the catalytic activity, the most remote mutations enhanced the enantioselectivity, and double mutations combined the two. Eventually, after pH optimization with an appropriate buffer, (*R*)-1-phenylethanol was obtained in 85% *ee* and 90% conversion after 64 hours (S/C = 100, 55 °C). It needs to be emphasized that the induction of chirality was largely brought about by protein-substrate and protein-complex interactions.

In 2006 [137], the Ward group continued the development of this intriguing hydrogenation system and tested 20 forms of streptavidin against 21 modifications of Ru^II^ complexes in the ATH of three different substrates at once. In the first instance, to accelerate the screening process, they screened only two forms of streptavidin with all 21 Ru catalysts. Based on the results, five catalysts were selected and further tested with 20 streptavidin isoforms. With this extensive pool of data, the authors confirmed that η^6^-*p*-cymene was pro-(*R*) and η^6^-benzene mainly pro-(*S*), although in the latter case it could be reversed to pro-(*R*) when certain mutations in streptavidin were applied. As a result, the most promising combinations of biotinylated complexes with particular streptavidin mutants were identified, and evaluated in the ATH of several ketone substrates. Furthermore, docking studies formed a significant part of this work, as they gave a valuable insight on the structure of the artificial metalloenzymes.

There has been a constant development in the area of Ward’s biotin-streptavidin species. Remarkably, one variant of their artificial hydrogenases, identified by employing a designed evolution protocol, was shown to reduce dialkyl ketones with up to 90% *ee* [138]. It has always been a great challenge to reduce such substrates with high enantioselectivity, since aromatic ketones facilitate the enantiodiscrimination by their disposition for the formation of a CH/π interaction with the η^6^-arene [77], which cannot be expected in the case of aliphatic ketones. Very recently, the concept was also applied to a cyclic imine [140], although with an iridium atom instead of ruthenium. Upon optimizing the reaction conditions, (*R*)-salsolidine was obtained in 96% *ee* and (*S*)-salsolidine could be synthesized in 78% *ee*.

A very interesting paper by Polborn and Severin [141] also belongs primarily in this section, in spite of the fact that it is based on immobilization of modified (η^6^-arene)RuCl(ethylenediamine) **26**. This is because their work goes beyond ordinary immobilization and seeks to attain substrate specificity *via* molecular imprinting (see also [142]) by a product-like molecule. As the authors wished to selectively hydrogenate benzophenone on this novel catalyst, the diphenylphosphinato ligand was selected for the coordination to the Ru atom as a pseudosubstrate. This association complex was then copolymerized with ethylene glycol dimethacrylate (EGDMA). Finally, the diphenylphosphinato ligands were selectively removed and re-substituted by chloro ligands, which created a highly substrate-specific molecularly-imprinted cavity adjacent to the catalyst active site. The molecular imprinting led to significant acceleration of the ATH of benzophenone, which proceeded with notable substrate selectivity when more ketones were present in the mixture. The complex was also found to be regioselective to diarylketone groups in more complex substrates.

Weng, Tada and co-workers [143] used this concept as well, but they performed the immobilization of modified **1b** (*N*-*p*-styrenesulfonyl-DPEN instead of TsDPEN, according to [100]) on functionalized solid SiO_2_. The molecular imprinting was carried out by the product molecule in question. Hence, after anchoring the catalyst, (*R*)-1-(*o*-fluorophenyl)ethanol (*i.e.*, the product of ATH of *o*-fluoroacetophenone) was coordinated onto the complex as a template. This association complex, representing a monomeric unit, was subject to stacking of surface matrix overlayers. This was performed by four different methods, out of which photopolymerization was eventually utilized in the case of the catalytic species used for subsequent testing in ATH. The final step was the removal of the template from the immobilized complex, which formed the molecularly-imprinted cavity. The novel solid catalyst was thoroughly characterized by analytical methods and the heights of polymer matrices were determined, locating the optimal height for the hydrogenation process (2 nm). Eight different substrates were tested using this catalyst with HCOONa in water with SDS, and the results were promising. The reaction rate was indeed highest in the case of *o*-fluoroacetophenone (except for 2-acetylfuran which was apparently very compatible with the imprinted cavity) and the corresponding (*R*)-1-(*o*-fluorophenyl)ethanol was obtained with 91% *ee*, which was a considerably higher value than the previously reported one [106].

## 4. Mechanistic Considerations

For every chemical reaction it is highly desirable to have a deep understanding of its mechanism. In the case of asymmetric reactions, it is even more important as it provides the rationale for the enantiofacial discrimination which is not always a trivial issue. In this text, we focus on the reaction mechanism of asymmetric transfer hydrogenations on complexes **1**.

### 4.1. ATH of Ketones Catalyzed by ***1*** in the Presence of Isopropanol and a Strong Base

**Scheme 1:**
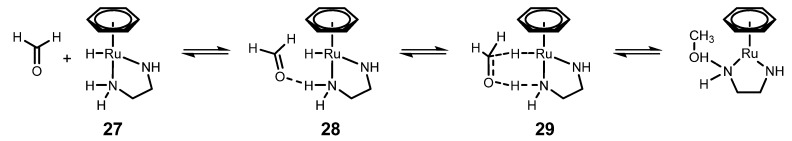
The original metal-ligand bifunctional mechanistic concept [144].

In 2000 [144], Noyori *et al.* published a seminal work where the fundamental features of the ATH of ketones using **1**, coined as metal-ligand (M-L) bifunctional catalysis, were proposed. The initial calculations upon this reversible system were performed using formaldehyde as a substrate, and simplified structures of the catalytic complexes, *i.e.*, RuH(η^6^-benzene)(ethylenediamine) **27** or RuH(η^6^-benzene)(ethanolamine), were used (Scheme 1). According to these findings, the substrate does not coordinate directly to the Ru centre, but forms a C=O**^…^**H-N hydrogen-bonded intermediate **28** with the ruthenium complex, which evolves into a six-membered cyclic transition state (TS) **29**. The hydrogen bond is supported by a so-called “NH effect”: the N-H bond in the chelating ligand is markedly more polar when in a complex with ruthenium. Thus, the six-membered TS results in a transfer of the proton from the NH group to the carbonyl oxygen, and a hydride transfer from Ru onto the C=O carbon atom. This novel mechanism was also compared with a *β*-elimination/insertion mechanism, which was shown to be energetically and sterically unfavoured.

**Scheme 2:**
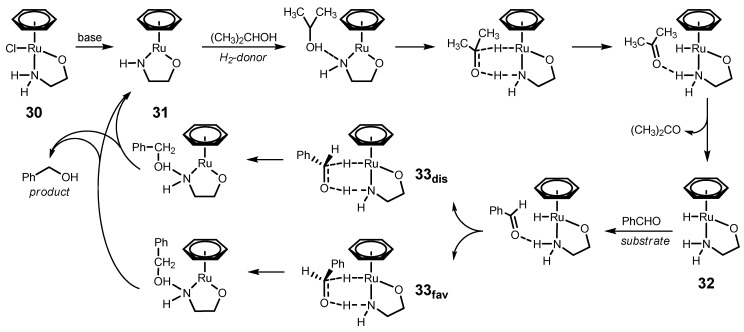
Metal-ligand bifunctional mechanism: ATH of benzaldehyde using **30** and *i*-PrOH [61].

The M-L bifunctional mechanism was further studied in the case of ATH of benzaldehyde using RuCl(η^6^-benzene)(ethanolamine) **30** and isopropanol as a hydrogen donor (Scheme 2) [61]. The Ru-hydride **32** conveys a proton and a hydride to the C=O bond of benzaldehyde, which affords the alcohol product and a 16e unsaturated Ru complex **31** [65]. Two TSs **33_fav_** and **33_dis_** are considered for the hydrogen transfer, one of which (**33_fav_**) has lower energy due to the CH/π interaction (*vide infra*). However, benzaldehyde is not a prochiral compound and thus both TSs yield benzyl alcohol. The 16e complex **31** is then regenerated to **32** through a hydrogen transfer from isopropanol, with acetone formed as a by-product. The Ru-hydride molecule **32** always arises from the 16e complex **31**, which is initially formed from the Ru-Cl precursor **30** by elimination of HCl by a strong base like KOH or *t*-BuOK.

The other important mechanistic aspect is the enantioselectivity of ATH. In this hydrogenation system, several factors evoke the asymmetric bias: (1) Employing the chiral chelating ligands like TsDPEN makes Ru complexes **1** chiral at the Ru atom, which is an essential prerequisite for conducting asymmetric reactions with these complexes. The configuration of the chelate is responsible for the configuration of the major product enantiomer [61,76,144]. When an (*S*,*S*)-diamine is used, the configuration at Ru is *R*, and (*S*)-1-phenylethanol is afforded preferentially in the ATH of acetophenone. With an (*R*,*R*)-diamine, the configuration at Ru is *S*, and (*R*)-product is obtained accordingly; (2) The η^6^-arene ligand is capable of a weak, attractive CH/π interaction with the aromatic ring of the substrate molecule [77]. This, among others, explains why aromatic ketones and imines are reduced by these complexes much more easily than aliphatic substrates. The CH/π attraction is the reason why two TSs of different energies are formed, as shown in Scheme 9. The TS characterized by lower energy (denoted as favourable TS or favTS) is formed with higher probability and leads to the desired product enantiomer, whereas the less probable TS (called disfavourable TS or disTS) affords the unwanted product enantiomer. FavTS is stabilized by the CH/π interaction and for that reason, it is energetically more probable. The character of the CH/π interaction can be affected by the selection of the η^6^-arene ligand. When η^6^-benzene is used, C(sp^2^)H/π attraction takes place, which is different from the C(sp^3^)H/π interaction in the case of e.g., η^6^-hexamethylbenzene.

Therefore, the hydrogenation proceeds in the outer coordination sphere of the ruthenium atom, where the chelating ligand plays a pivotal role. According to Clapham’s classification of hydrogenations catalyzed by Ru-hydride complexes, this mechanism belongs to the TOL group [45].

### 4.2. ATH Catalyzed by ***1*** in the Presence of the HCOOH/Triethylamine Azeotrope

**Scheme 3:**
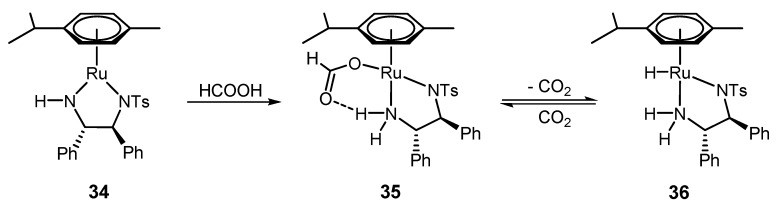
Structures observed in the reaction mixture when HCOOH/triethylamine is used in ATH.

A great deal of ATHs using **1** are carried out in the presence of the HCOOH/triethylamine azeotrope. Utilizing formic acid entirely changes the reaction behaviour, rendering it almost irreversible because gaseous CO_2_ is formed as a by-product. This is in a marked contrast to the reductions using isopropanol as a hydrogen source, where the resulting acetone can react in the reverse direction and cause the overall reversibility. However, CO_2_ needs to be effectively removed by stirring or N_2_-bubbling in order to reach highest reaction rates and the desired irreversibility [145] as it is assumed that it can form a ruthenium-formate complex **35** by insertion into the Ru-H bond of **36** (Scheme 3) [146], which is not favoured in this context.

In this case, the reaction mechanism has not yet been fully understood. In addition to the Ru-hydride **36** and the 16e complex **34**, a Ru-formate species **35** is formed at the beginning of the reaction from HCOOH and **34** [146]. At low temperatures, only **35** was observed by NMR, while raising the temperature led to decarboxylation and formation of **36** [147]. Therefore, **36** is thought to arise from **35**, which is formed first.

### 4.3. Mechanism of ATH Catalyzed by ***1*** in Water

As this review has extensively dealt with ATH of ketones and imines in aqueous media, some mechanistic considerations on these reactions are presented here. The group of Xiao recently published an in-depth study on the ATH of acetophenone conducted in water with **1b** and HCOONa [148]. They arrived at a number of very interesting conclusions, promoting the role of H_2_O from a mere solvent to a highly important, actively involved species. It was found to facilitate the decarboxylation of **35** to **36**, rendering the Ru-hydride **36** formation very fast with **35** not observable by NMR. The 16e complex **34** is supposedly stabilized by the water molecule *via* a transformation into Ru-aqua or Ru-hydroxyl species which were observed by NMR at −80 °C. Perhaps the most striking conclusion is the active participation of water in the TSs which was deduced from density functional theory (DFT) calculations. The water molecule is assumed to hydrogen-bond to the oxygen atom of the ketone C=O group, which lowers the energy of the TS by ~4 kcal mol^–1^, and thus facilitates hydrogen transfer. Furthermore, the catalytic cycles significantly differ under acidic, basic and neutral conditions, the latter of which are favoured. Acidic conditions severely decrease both the reaction rate and enantioselectivity as a result of the partial decoordination of the TsDPEN ligand through protonation of its amido nitrogen [60]. 

We believe that these selected mechanistic aspects demonstrate the importance of water in the mechanism of ATH conducted in aqueous solutions. The positive effects introduced by the H_2_O molecule to the reaction mechanism further support its use instead of traditional organic media.

### 4.4. ATH of Imines Catalyzed by **1** in the Presence of the HCOOH/Triethylamine Azeotrope

**Scheme 4:**
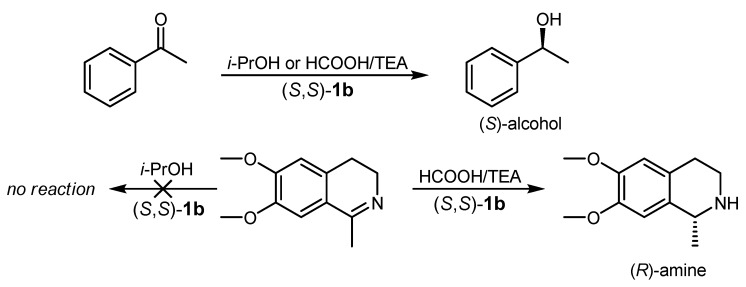
Major differences in ATH of ketones and imines using the catalyst (*S*,*S*)-**1b**.

Initially, the stereoselective transfer hydrogenations of imines were carried out with HCOOH/triethylamine as isopropanol turned out to be completely ineffective [53]. Recently, it has been found that imines can only be reduced under acidic conditions [149], which conforms to the inapplicability of isopropanol as a hydrogen source. However, inherent imine protonation is not compatible with the original mechanism described in Section 4.1. In 2009, Wills and co-workers discussed all possible favTSs for the ATH of 6,7-dimethoxy-1-methyl-3,4-dihydroisoquinoline catalyzed by **1** [82] which created a further discrepancy with the original mechanism with regard to the absolute configuration of the product of ATH (Scheme 4). If the standard six-membered favTS structure were applied with (*S*,*S*)-**1b**, the outcome [(*S*)-amine] would be a different enantiomer from the one observed experimentally, *i.e.*, (*R*)-amine. This evidence suggests that a different pathway should be considered for the ATH of imines. This issue is further addressed in Section 4.5.

### 4.5. Molecular Modelling

Modelling the intermediates and TSs *in silico* grants us very specific information on their geometries. The M-L bifunctional mechanism was elucidated by performing high-precision *ab initio* calculations [61,77,144]. These computational methods are capable of providing 3D models which tell us much about the steric properties of a given compound. In addition, the energy values obtained can help us predict for example thermodynamic properties, reactivity, or even enantioselectivity [150].

In our recent paper [151], we proposed the TS structures for the ATH of acetophenone (a ketone) and 1-methyl-3,4-dihydroisoquinoline (an imine) catalyzed by (*S*,*S*)-**1b**. This imine molecule was selected for its simplicity since all ruthenium complex structures were treated “as is” without any simplifications, which resulted in very high hardware demands. All calculated TSs conformed to the product configurations observed experimentally. (*R*)-1-methyl-1,2,3,4-tetrahydroisoquinoline and (*S*)-1-phenylethanol were thus obtained as the outcomes from favTSs. 

**Figure 8:**
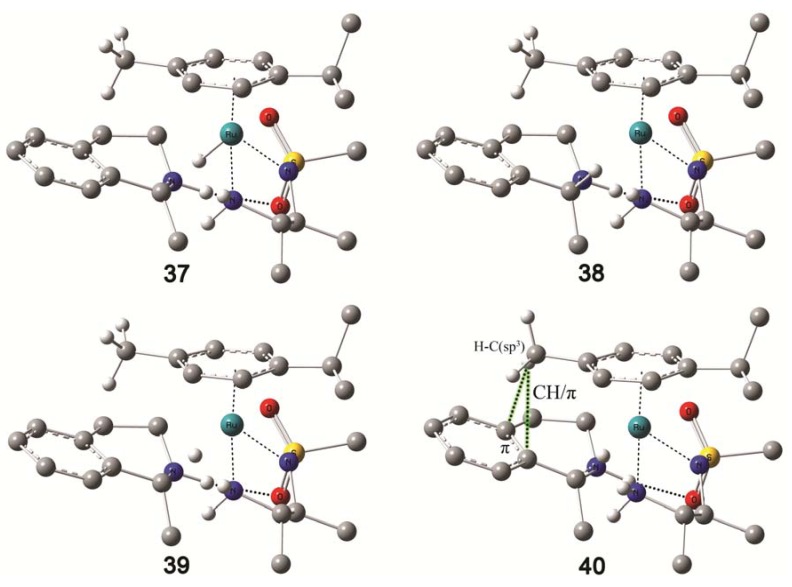
Cut-off input geometries for TS optimization. The C(sp^3^)H/π interaction is shown in green. The unnecessary hydrogen atoms have been omitted for clarity.

All calculations were performed at the density functional theory (DFT) level, using the B3LYP [152,153] hybrid functional. For all atoms except ruthenium, the 6-31G(d,p) basis set was used; ruthenium was treated with the LANL2DZ basis set and effective core potential (ECP) [154]. The initial Ru-hydride model **36** was obtained by optimizing the X-ray coordinates of **1b** published by Noyori in 1996 [53], where the chlorine atom was substituted by a hydrogen atom. As all other models were constructed and optimized “from scratch”, finding the TS structures was a rather difficult task. Optimizations of the TSs (Figure 8) were performed by the QST3 method, where three input geometries need to be specified: the reactant (**37**), the product (**38**) and an initial guess of the TS structure (**39**). Successful convergence of a TS optimization process is highly sensitive and the initial guesses need to be very precise. For that reason, the screening was accelerated by utilizing cut-off geometries of the Ru complex formed by replacing all three aromatic rings of TsDPEN with methyl groups. This operation significantly reduced the number of atoms present and the calculations were thus much faster, which allowed us to encounter the TS geometries more promptly. The optimized cut-off TSs (e.g., **40** in Figure 1) were then rebuilt into full-scale structures and used for the real TS optimizations.

**Figure 9:**
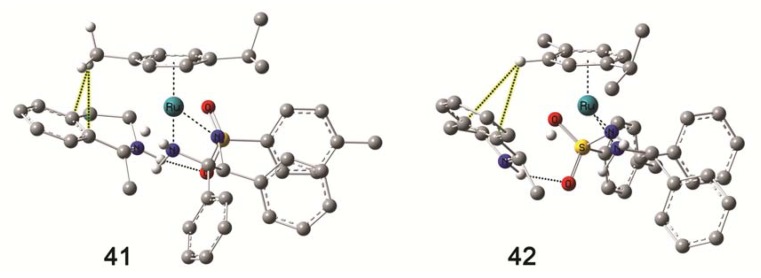
Optimized structures of favTSs utilizing C(sp^3^)H/π (**41**) or C(sp^2^)H/π (**42**) interactions (shown in green). The unnecessary hydrogen atoms have been omitted for clarity.

In this way we obtained the full-scale TS structures (**41**,**42**) depicted in Figure 9. The C(sp^3^)H/π (for **41**) and C(sp^2^)H/π (**42**) attractions are depicted in green. It can be seen that the imine is *N*-protonated and only one hydrogen is transferred from Ru-H to the carbon atom of the C=N bond. The (*S*,*S*)-ligand leads to the (*R*) configuration of the amine product. Another important attraction, which has not been described before, was revealed between the =NH^+^- group of the substrate and the O=S fragment of TsDPEN ligand. It was found to significantly contribute to the stabilization of TSs.

However, it was necessary to explore in detail which CH/π attractive forces (*i.e.*, C(sp^2^)H/π or C(sp^3^)H/π) were exploited in this particular reaction. This was due to the use of η^6^-*p*-cymene in our calculations, which hypothetically allowed both options. The symmetry of the *p*-cymene ring allows four different sites which can be used in a CH/π attraction. Out of these, two were shown to be probable, one of them being C(sp^3^)H/π and the other C(sp^2^)H/π (in **41** or **42**, shown in Figure 2).

To sum up, imines apparently cannot be reduced without the necessary *N*-protonation, which can occur *in situ* e.g., in the HCOOH/triethylamine H_2_-donor mixture. Upon hydrogenation by complexes **1**, a different product configuration is observed while only ketones conform to the original M-L bifunctional mechanism. In the case of imines, different structures of the TSs have been suggested and they are in agreement both with the substrate protonation and the absolute configuration of the product.

## 5. Conclusions

The purpose of this review was to give an account on the group of Noyori’s renowned η^6^-arene/*N*-arylsulfonyl-1,2-diphenylethylenediamine-ruthenium(II) catalysts **1**. The catalysts were discussed in the context of their immobilization, recyclability, use in aqueous media and other specific applications which very often bring about notable improvements. Although the original catalysts **1** could be used in some situations of this kind, most of the work dealt with slight modifications of the catalyst structure. Frequently, this led to enhancements in the catalytic activity, chemo-, regio- and enantio-selectivity, reusability, or extended the substrate scope.

It can be seen that this catalytic system for the ATH of ketones and imines is already very well established. The reaction mechanism has been largely characterized, which allows for precise optimization of reaction conditions for a given substrate. However, some aspects are still shrouded in secrecy, especially when considering the ATH of imines using the HCOOH/triethylamine azeotrope as a hydrogen donor.

## Figures and Tables

**Table 1:** The basic set of η^6^-arene/*N*-sulfonyldiamine-Ru^II^ catalysts and their representative reactions.

Entry	Catalyst	Substrate	Product	S/C ^[a]^; Time; Temp.; Conversion (%)	% *ee* (config) ^[b]^	Ref. ^[c]^
1	**(*R*,*R*)-1a**	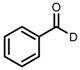	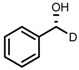	200; 0.5 h; 28 °C; 100	97 (*R*)	[26]
2	**(*S*,*S*)-1b**	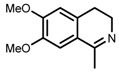	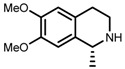	200; 3 h; 28 °C; 99	95 (*R*)	[53]
3	**(*R*,*R*)-1b** ^[d]^	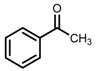	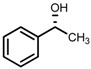	100; 20 h; 28 °C; > 99	97.7 (R)	[57]
4	**(*S*,*S*)-1c**	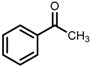	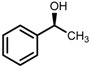	200; 20 h; 28 °C; 99	98 (*S*)	[54]
5	**(*S*,*S*)-1c** ^[d]^	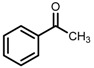	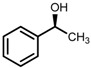	200; 15 h; r.t.; 99	97 (*S*)	[52]
6	**(*R*,*R*)-1d**	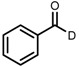	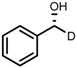	200; 7 h; 28 °C; 99	96 (*R*)	[26]
7	**(*S*,*S*)-1e**	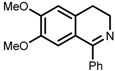	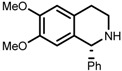	200; 8 h; 28 °C; 99	84 (*R*)	[53]
8	**(*R*,*R*)-1f**	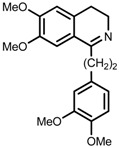	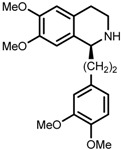	200; 12 h; 28 °C; 99	92 (*S*)	[53]

^[a]^ Molar ratio substrate/catalyst; ^[b]^ See corresponding references for details on determination of *ee* and product configuration; ^[c]^ Reference containing data for a given entry; ^[d]^ Catalyst was formed *in situ* from the [RuCl_2_(η^6^-arene)]_2_ dimer and the corresponding *N*-arylsulfonylethylene-1,2-diamine.

**Table 2:** ATH of acetophenone catalyzed by **1b** in water ^[a]^.

Entry	Catalyst config.	Solution	Time; Temp.; Conversion (%)	% *ee* (config) ^[b]^	Ref. ^[c]^
1	(*R*,*R*)	H_2_O/HCOONa	2 h; 40 °C; >99	94 % (*R*)	[59]
2	(*R*,*R*)	HCOOH/Et_3_N	12 h; 40°C; 98	97 % (*R*)	[59]
3	(*R*,*R*)	H_2_O/HCOONa/CTAB ^[d]^	4 h; 28 °C; >99	95 % (*R*)	[62]
4	(*S*,*S*)	H_2_O/HCOONa/PEG	15 h; 40 °C; >99	96 % (*S*)	[63]

^[a]^ Molar ratio substrate/catalyst (S/C) = 100; The catalyst was formed *in situ* from the [RuCl_2_(η^6^-*p*-cymene)]_2_ dimer and TsDPEN; ^[b]^ See corresponding references for details on determination of *ee* and product configuration; ^[c]^ Reference containing data for a given entry. ^[d]^ CTAB = cetyltrimethyl-ammonium bromide.

**Table 3:** ATH of acetophenone catalyzed by immobilized **1b**.

Entry	Catalyst config.	Substrate	Solution	S/C ^[a]^; Time; Temp.; Conversion (%)	% *ee* (config) ^[b]^	Ref. ^[c]^
1	(*S*,*S*) ^[d]^	DHIQ ^[e]^	HCOOH/Et_3_N; acetonitrile	200; 7 h; r.t.; 99	89% (*R*)	[67]
2	(*S*,*S*) ^[f]^	acetophenone	H_2_O/HCOONa; Et_4_N^+^Br	100; 5 h; 30 °C; >99	93% (*S*)	[68]

^[a]^ Molar ratio substrate/catalyst; ^[b]^ See corresponding references for details on determination of *ee* and product configuration; ^[c]^ Reference containing data for a given entry; ^[d]^ **1b** was immobilized in functionalized MCM-41; ^[e]^ DHIQ = 1-methyl-3,4-dihydroisoquinoline; ^[f]^ **1b** was immobilized in a nanocage of SBA-16.

**Table 4:** ATH of ketones and imines in water using modified diamine ligands ^[a]^.

Entry	Ligand	Substrate	Solution	Time; Temp.; Conversion (%)	% *ee* (config) ^[b]^	Ref. ^[c]^
1	(*S*,*S*)-**2**	acetophenone	H_2_O/*i*-PrOH/*t*-BuOK	48 h; 22 °C; 96	94 (*S*)	[91]
2	(*R*,*R*)-**3**	acetophenone	H_2_O/HCOONa; SDS ^[d]^	24 h; 40 °C; >99	95 (*R*)	[93]
3	(*R*,*R*)-**3**	*α*-bromo-acetophenone	H_2_O/CH_2_Cl_2_/HCOONa; SDS	24 h; 28 °C; 87 (isol. yield)	94	[93]
4	(*R*,*R*)-**3**	DHIQ ^[e]^	H_2_O/HCOONa; CTAB ^[f]^	10 h; 28 °C; 97 (isol. yield)	95 (*S*)	[94]
5	(*S*,*S*)-**4**	acetophenone	H_2_O/HCOONa	0.5 h; 28 °C; 33	95 (*S*)	[95]
6	(*R*,*R*)-**5c**	acetophenone	H_2_O/HCOONa	2 h; 40 °C; 100	94 (*R*)	[96]
7	(*R*,*R*)-**5d**	acetophenone	H_2_O/HCOONa	2 h; 40 °C; 100	92 (*R*)	[96]

^[a]^ Molar ratio substrate/catalyst (S/C) = 100; The catalyst was formed in situ from the [RuCl_2_(η^6^-*p*-cymene)]_2_ dimer and the corresponding ligand; ^[b]^ See corresponding references for details on determination of *ee* and product configuration; ^[c]^ Reference containing data for a given entry; ^[d]^ SDS = sodium dodecyl sulfate; ^[e]^ DHIQ = 6,7-dimethoxy-1-methyl-3,4-dihydroisoquinoline; ^[f]^ CTAB = cetyltrimethylammonium bromide.

**Table 5:** ATH of ketones and imines using immobilized derivatives of **1** ^[a]^.

Entry	Ligand	Substrate	Solution	S/C ^[b]^; Time; Temp.; Conversion (%)	% *ee* (config) ^[c]^	Ref. ^[d]^
1	(*S*,*S*)-**6b**	acetophenone	*i*-PrOH/KOH	20; 48 h; – ; 23	84 (*S*)	[100]
2	(*S*,*S*)-**7a**	acetophenone	HCOOH/Et_3_N/ CH_2_Cl_2_	100; 18 h; 30 °C; 71	> 99 (*S*)	[101]
3	(*S*,*S*)-**7b**	acetophenone	HCOOH/Et_3_N	100; 28 h; 30 °C; 95	96.7 (*S*)	[101]
4	(*R*,*R*)-**8**	acetophenone	HCOOH/Et_3_N	100; 20 h; 50 °C; 95	94 (*R*)	[102]
5	(*R*,*R*)-**8**	acetophenone	H_2_O/HCOONa	100; 1 h; 40 °C; 99	92 (*R*)	[103]
6	(*R*,*R*)-**9a**	acetophenone	HCOOH/Et_3_N	100; 6 h; 40 °C; >99	97 (*R*)	[104]
7	(*R*,*R*)-**9b**	acetophenone	H_2_O/HCOONa; TBAB ^[e]^	100; 2 h; 40 °C; >99	96 (*R*)	[105]
8	(*S*,*S*)-**10b**	2-cyano-acetophenone	HCOOH/Et_3_N	100; 17 h; 30 °C; 98 (isol. yield)	97 (*S*)	[107]
9	(*S*,*S*)-**11**	DHIQ ^[f]^	HCOOH/Et_3_N/CH_2_Cl_2_	100; 12 h; r.t.; 100 (isol. yield)	91 (*R*)	[108]
10	(*R*,*R*)-**12b**	acetophenone	H_2_O/HCOONa	100; 3 h; 40 °C; 100	98 (*R*)	[109]
11	(*R*,*R*)-**13b**	acetophenone	H_2_O/HCOONa	100; 3 h; 40 °C; 100	98 (*R*)	[109]
12	(*R*,*R*)-**13a**	DHIQ ^[f]^	H_2_O/HCOONa; CTAC ^[g]^	100; 24 h; r.t.; 69	94 (*S*)	[110]
13	(*S*,*S*)-**14**	acetophenone	H_2_O/HCOONa	100; 2 h; r.t.; >99	96 (*S*)	[111]
14	(*S*,*S*)-**15**	DHIQ ^[f]^	HCOOH/Et_3_N/CH_2_Cl_2_	100; 1.5 h; 40 °C; 99	94 (*R*)	[112]
15	(*R*,*R*)-**16**	acetophenone	H_2_O/HCOONa	100; 9 h; 40 °C; 100	97 (*R*)	[113]

^[a]^ The catalyst was formed in situ from the [RuCl_2_(η^6^-*p*-cymene)]_2_ dimer and the corresponding ligand; ^[b]^ Molar ratio substrate/catalyst; ^[c]^ See corresponding references for details on determination of *ee* and product configuration; ^[d]^ Reference containing data for a given entry. ^[e]^ TBAB = tetrabutylammonium bromide; ^[f]^ DHIQ = 6,7-dimethoxy-1-methyl-3,4-dihydroisoquinoline; ^[g]^ CTAC = cetyltributyl-ammonium chloride.

**Table 6:** ATH of acetophenone using dendritic catalysts ^[a]^.

Entry	Ligand	Solution	Time; Temp.; Conversion (%)	% *ee* (config.) ^[b]^	Ref. ^[c]^	
1	(*S*,*S*)-**17** ^[d]^	HCOOH/Et_3_N	20 h; 28 °C; >98	96.5 (*S*)	[125]	
2	(*R*,*R*)-**18a**	HCOOH/Et_3_N	20 h; 28 °C; 99	97.6 (*R*)	[57]	
3	(*R*,*R*)-**19**	HCOOH/Et_3_N	20 h; 28 °C; 97	97.1 (*R*)	[57]	
4	(*R*,*R*)-**20** ^[e]^	HCOOH/Et_3_N	20 h; 28 °C; 97	97.1 (*R*)	[128]	
5	(*R*,*R*)-**21** ^[f]^	HCOOH/Et_3_N/CH_2_Cl_2_	20 h; 28 °C; 97	96.1 (*R*)	[129]	
6	(*S*,*S*)-**17** ^[e]^	*i*-PrOH/*i*-PrOK	48 h; 25 °C; 65	95 (*S*)	[130]	
7	(*S*,*S*)-**22**	H_2_O/HCOONa; TBAI ^[g]^	4 h; 40 °C; > 99	97 (*S*)	[131]	

^[a]^ Molar ratio substrate/catalyst (S/C) = 100; The catalyst was formed in situ from the [RuCl_2_(η^6^-*p*-cymene)]_2_ dimer and the corresponding ligand; ^[b]^ See corresponding references for details on determination of *ee* and product configuration; ^[c]^ Reference containing data for a given entry; ^[d]^ For all generations (*n* = 1-3), similar performances were observed; ^[e]^ Data for the third generation (*n* = 3); ^[f]^ Data for the second generation (*n* = 2); ^[g]^ TBAI = tetrabutylammonium iodide.

**Table 7:** ATH of acetophenone performed in ionic liquids.

Entry	Ligand	Solution	S/C ^[a]^; Time; Temp.; Conversion (%)	% *ee* (config.) ^[b]^	Ref. ^[c]^
1	(*R*,*R*)-**23** ^[d]^	HCOOH/Et_3_N/[C_4_C_1_C_1_Im]PF_6_^[e]^	200; 24 h; 35 °C; > 99	> 99	[132]
2	(*S*,*S*)-**24** ^[f]^	HCOOH/Et_3_N/[bmim][PF_6_] ^[g]^	100; 24 h; r.t.; 98	92 (*S*)	[72]
3	(*R*,*R*)-**5a** ^[h]^	HCOOH/Et_3_N/[bmim][PF_6_] ^[g]^	100; 8 h; 40 °C; 100	97 (*R*)	[99]

^[a]^ Molar ratio substrate/catalyst; ^[b]^ See corresponding references for details on determination of *ee* and product configuration; ^[c]^ Reference containing data for a given entry; ^[d]^ The catalyst was formed *in situ* from (*R*,*R*)-TsDPEN and the corresponding ligand; ^[e]^ 1-butyl-2,3-dimethylimidazolium hexafluoro-phosphate; ^[f]^ The catalyst was formed *in situ* from the [RuCl_2_(η^6^-benzene)]_2_ dimer and (*S*,*S*)-**24**; ^[g]^ [bmim][PF_6_] = 1-butyl-3-methylimidazolium hexafluorophosphate; ^[h]^ The catalyst was formed *in situ* from the [RuCl_2_(η^6^-*p*-cymene)]_2_ dimer and (*R*,*R*)-**5a**.
